# Enhanced vapor sorption in block and random copolymer brushes†

**DOI:** 10.1039/d2sm00868h

**Published:** 2022-10-08

**Authors:** Ivona Glišić, Guido C. Ritsema van Eck, Leon A. Smook, Sissi de Beer

**Affiliations:** Sustainable Polymer Chemistry Group, Department of Molecules & Materials, MESA+ Institute for Nanotechnology, University of Twente, P.O. Box 217 7500 AE Enschede The Netherlands s.j.a.debeer@utwente.nl +31 53 4893170

## Abstract

Polymer brushes in gaseous environments absorb and adsorb vapors of favorable solvents, which makes them potentially relevant for sensing applications and separation technologies. Though significant amounts of vapor are sorbed in homopolymer brushes at high vapor pressures, at low vapor pressures sorption remains limited. In this work, we vary the structure of two-component polymer brushes and investigate the enhancement in vapor sorption at different relative vapor pressures compared to homopolymer brushes. We perform molecular dynamics simulations on two-component block and random copolymer brushes and investigate the influence of monomer miscibility and formation of high-energy interfaces between immiscible monomers on vapor sorption. Additionally, we present absorption isotherms of pure homopolymer, mixed binary brush and 2-block, 4-block, and random copolymer brushes. Based on these isotherms, we finally show that random copolymer brushes absorb more vapor than any other architecture investigated thus far. Random brushes display enhanced sorption at both high and low vapor pressures, with the largest enhancement in sorption at low vapor pressures.

## Abstract 2

Copolymer brushes in gaseous environments absorb and adsorb vapors of favorable solvents.

## Graphical abstract

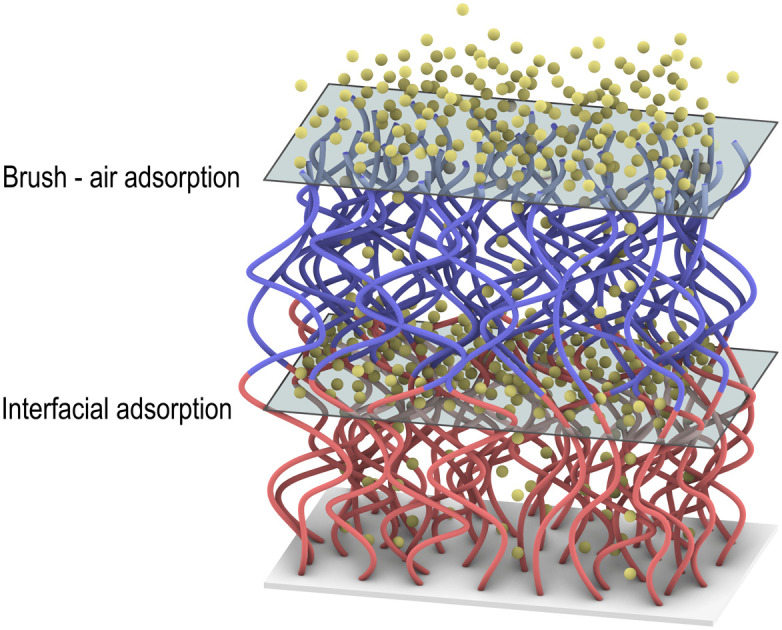

## 1 Introduction

Polymer brushes consist of long macromolecules that are attached to a substrate at a density that is high enough for the polymers to stretch away from the substrate.^1^ These brushes can be utilized in a plethora of applications, such as smart adhesives,^2,3^ sensors,^4–6^ nanofluidics^7,8^ and membrane technologies.^9,10^ While early research focused on applying polymer brushes in liquid, it was recently recognized that brushes can be employed broadly in air as well.^11^ For example, lubricants,^12^ vapor sensors,^13,14^ moisture harvesters^15^ or gas separation technologies^16,17^ can benefit from brush functionalization, because the brushes can absorb vapor from the air. For most of these applications it is important that vapor sorption in the brush is maximized. However, this is difficult to achieve, especially at low vapor pressures.

The reason for the typical low absorption at low vapor pressures can be understood as follows. The amount of vapor absorbing in a brush is strongly affected by the vapor pressure^18–24^ and the isotherm describing this can be determined by an extended version of the Flory–Huggins theory,^25,26^ as proposed by Birshstein and Lyatskaya.^27^ The exact shape of these isotherms depends on the brush parameters (grafting density and chain length) and the solvent quality. However, in most experiments the isotherms are observed to be concave-upward,^18–24^ with minimal absorption at low vapor pressures and a strong increase in absorption only near the saturation pressure of the vapor. This means that at low vapor concentrations, vapor sorption in brushes is typically very limited, unless alternative strategies are being employed.

In a recent publication, we have shown that vapor sorption at low concentrations can be strongly increased by utilising binary brushes composed of immiscible polymers (A and B).^28^ These immiscible polymers can phase separate in nano-domains and excess vapor adsorbs at the high-energy polymer–polymer interface. This can, depending on the brush characteristics,^29^ result in the sorption at low concentrations being even a factor 10 higher compared to sorption in homopolymer brushes. The best performance was observed for high density brushes with equal fractions of A and B polymers. Yet, these binary brushes are difficult to obtain synthetically.^30^ Brushes with equal A–B fractions can be obtained by triblock copolymers, which are grafted by their middle block to the substrate to form y-shaped binary polymer structures.^31,32^ However, due to steric hindrance, the grafting density for these structures will be rather low. And while high density binary brushes can be obtained by grafting-from strategies,^33^ for example from mixed monolayers with initiators for two different polymerization reactions^34^ which allows for the consecutive polymerization of the A and B polymers, it is difficult to obtain equal fractions of both polymers in these systems. Therefore, we need polymer brush systems with two components that introduce high-energy interfaces and are easy to synthesize.

Block copolymers of incompatible polymers are promising candidates for producing high-energy interfaces in brushes with relative ease. Coatings of incompatible block copolymers have been extensively researched for their ability to spontaneously phase-separate into nanometer-scale domains, which finds potential applications in lithography.^35^ Block and random copolymers of poly(styrene-*co*-(methyl methacrylate)), an archetypical incompatible copolymer, can be synthesized by a variety of methods including living anionic polymerization,^36,37^ nitroxide-mediated radical polymerization,^36,37^ and click reactions between end-functionalized homopolymers.^38^ Many of these polymerization methods can also be initiated from functionalized surfaces,^33,39^ making the synthesis of high-density brushes of incompatible copolymers feasible. Additionally, extremely incompatible (“high-*χ*”) block copolymers are an active topic of research.^40,41^

To explore the feasibility of copolymer systems for vapor sorption, we employ molecular dynamics simulations to study the sorption enhancement of 2-block, 4-block and random copolymer brushes relative to a pure homopolymer brush. Additionally, we vary the interaction between the monomer species, and investigate how this influences phase separation and sorption in the brush. Finally, we vary the solvent vapor concentration to obtain absorption isotherms for all the aforementioned structures, as well as the previously studied mixed homopolymer brushes.

## 2 Models and methods

To study the solvent distribution in a variety of binary polymer brush systems, we use coarse-grained molecular dynamics simulations. Such coarse-grained simulations are suitable for studying general scalings and trends in materials and microscopic systems, such as polymers^42^ or functional brushes^12,43,44^ and gels.^45^ In this work, we simulate a variety of polymer brushes under implicit poor solvent conditions to model the conditions in dry air, and we expose them to an explicit good solvent to simulate the sorption of a solvent vapor.

**Fig. 1:**
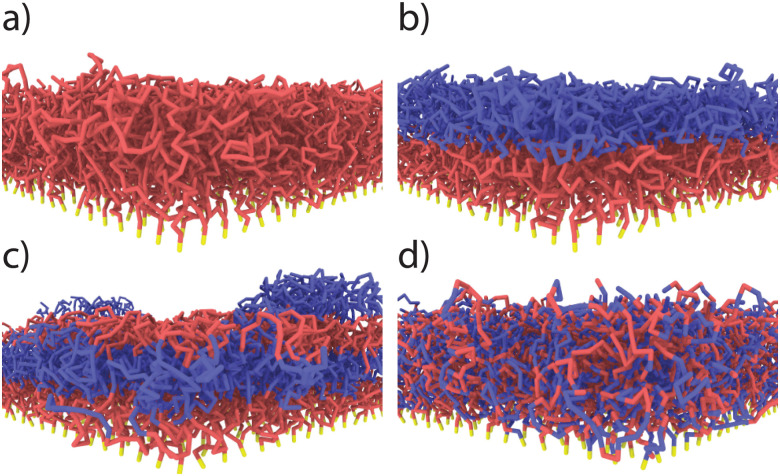
Illustrations of: (a) pure homopolymer brush, (b) 2-block brush, (c) 4-block brush, (d) random brush.

We create Kremer–Grest polymer brushes by grafting the polymers to the substrate *via* an anchoring point (*z* = 0). Monomer beads are stacked on top of each other perpendicular to the grafting surface. While a pure brush consists of a single monomer type, binary brushes consist of two immiscible monomer types: monomer A (dark, purple, near grafting plane) and monomer B (light, blue) (see Fig. 1). Besides a homopolymer brush (Fig. 1a), three different mixed brushes are created with a chain length of 32 beads: a 2-block copolymer brush (Fig. 1b), a 4-block copolymer brush (Fig. 1c), and a random copolymer brush (Fig. 1d). In each brush, monomer A is grafted closer to the grafting surface, and the length of block segments is equal. In other words, in a 2-block brush, we build two blocks of monomer A and monomer B each consisting of 16 monomers. Similarly, in a 4-block (ABAB) each of the four blocks consists of 8 monomers. Finally, the random brush is created by randomly substituting 50% of monomers of a homopolymer brush by the other monomer type. Grafting density is kept constant in all brushes at 0.25 *σ*^−2^ (critical grafting density *ρ*_g,*_ = 0.1263 *σ*^−2^). The modeled system consists of a rectangular box 40 × 40 × 80 *σ*^3^ in the *x*, *y*, and *z* directions, respectively, with a periodic boundary condition in the *x* and *y*-direction and a fixed boundary condition in *z*-direction. The fixed boundary conditions in *z* are enforced by a repulsive harmonic wall potential whose spring constant is set to 100 *εσ*^−2^ to prevent vapor and polymer particles from escaping from the simulation box.

Interactions between the particles in the system are described by two different potentials for bonded and non-bonded particles. The Lennard-Jones (LJ) potential (eqn (1)) is used to simulate non-bonded interactions, where *U* is the potential, *ε* the depth of the potential well, *r* the distance between two particles, and *σ* the zero-crossing distance.^46,47^1
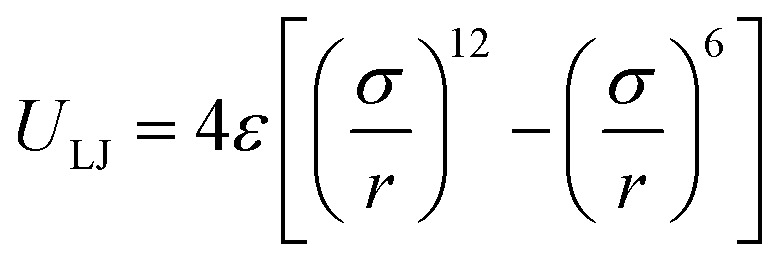
2
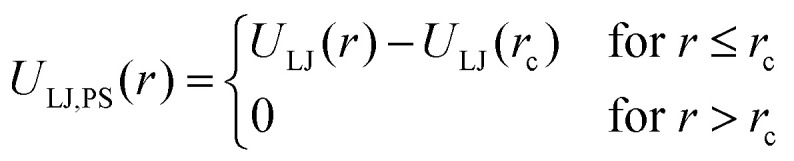
The expression has an energetic minimum at 
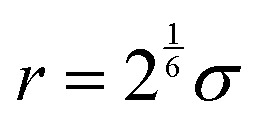
. The specific form of the LJ potential used in our simulations is truncated and potential shifted (PS) where *r*_c_ is the cut-off (here *r*_c_ = 2.5 *σ*). By varying *ε*, the depth of the energy well in the LJ potential is changed. Thus, the strength of the interactions between polymer–polymer (*ε*_pp_ = *ε*_aa_ = *ε*_bb_), monomer A–monomer B, (*ε*_ab_), solvent–solvent (*ε*_ss_), and polymer–solvent (*ε*_ps_ = *ε*_as_ = *ε*_bs_) can all be changed individually. Here the subscripts refer to the particle type: a – monomer type A, b – monomer type B, p – any monomer type, s – solvent/vapor. Since all particles in our system are the same size, we use *ε* for particle–particle interactions. If the particles were not the same size, we would have to account for the particle size effect in terms of the virial coefficient.^48^

The bonded interactions are modeled by combining finite extensible nonlinear elastic (FENE) and Weeks–Chandler–Anderson (WCA) potential (eqn (3)). Bonded interactions are described by the sum of both the FENE and WCA potential (eqn (5)).^46,47^3
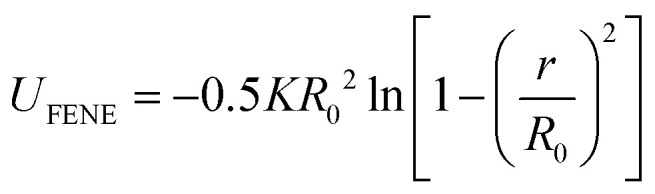
4
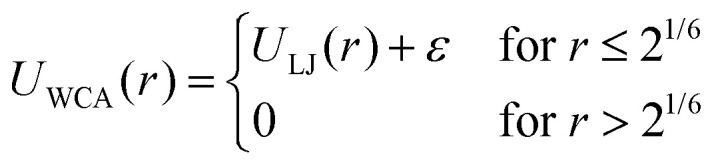

5 *U*_bond_ = *U*_FENE_ + *U*_WCA_

Here, *K* is the spring constant and *R*_0_ the maximum bond length. In the simulations the parameters are set to *K* = 30 *εσ*^−2^, *R*_0_ = 1.5 *σ*, *ε* = 1 and *σ* = 1. These values prevent the polymer chains from crossing each other and nonphysical behavior related to chain dynamics is prevented.^46,47^

The timesteps between the Grand Canonical Monte Carlo (GCMC) steps (described later) are simulated in the canonical ensemble (*NVT*). The system is thermostatted to a temperature of 0.85 *εk*_B_^−1^ by a chain of three Nosé–Hoover thermostats and the damping constant *τ*_d_ is set to 0.15 *τ*, where *τ* is a reduced unit for time. We use the rRESPA multi-scale integrator^49^ to allow the use of different timesteps for different interactions to speed up time integration. The time steps were chosen to be 0.0075 and 0.015 *τ* for the bonded interactions and the non-bonded interactions, respectively. All simulations are performed in LAMMPS.^50^

Each simulation consists of three steps: energy minimization, equilibration and production. First, we use an energy minimization on the artificial system so that the equilibration starts at a low-energy state. Then, we equilibrate the system in two steps. In the first step, a short *NVT* run (5 × 10^4^ time steps) is computed where particle displacements are limited, and then a longer run (5 × 10^5^ time steps) is performed where this limitation is lifted. We confirm that equilibrium is reached by observing that polymer density profiles no longer change with time. After the equilibrium is reached, we start the production run (3 × 10^6^ time steps) where the vapor is introduced to the system *via* GCMC method (constant *μVT*).^26,28^ During this production run, particle density profiles and snapshots are generated. A particle density profile normal to the grafting plane is computed every 10^5^ timesteps by averaging over the final 100 configurations of this window at intervals of 100 timesteps. Snapshots are visualized in OVITO.^51^

In our GCMC simulation set-up, the algorithm attempts to insert or remove vapor particles from a virtual reservoir into the simulation box every 10^4^ steps. The insertions and deletions are evaluated using the Metropolis criterion. Since the GCMC assumes ideal gas behavior while the LJ vapor in the box shows non-ideal behavior, we compensate for this non-ideality using previously found correlations between the imposed and actual vapor pressure in the simulation.^26^

Similar to previous work,^26^ we use the inflection point of the total polymer density profile (A + B) as a measure for the height of a polymer brush. Next, we integrate the density profiles below the brush height to find the solvent and polymer content in the brush, which are then converted into a solvent fraction. Any solvent above the brush height, including the adsorption film, is excluded from consideration so that interfacial effects between brush and vapor do not affect our solvent fraction.

We perform three sets of simulations.

1. Brush architecture. To see the effect of the polymer architecture, we expose a pure, 2-block, 4-block and random brush to an explicit good solvent and determine the local solvent fraction for each of them. Here, we set *ε*_ab_ to 0.4, *ε*_pp_ to 1, *ε*_ss_ to 1, and *ε*_ps_ to 1. Under these conditions, the solvent does not have any preference for a certain monomer type and the different polymers are expected to separate into different phases.^29^ These simulations are performed at a constant relative pressure *P*/*P*_sat_ ≈ 0.619. Based on these simulations, we determine which brush sorbs the most solvent.

2. Interfacial effect. To find the effect of the interface on vapor sorption, we use the same interaction strengths and vapor pressure as in the first set, except we vary the polymer cross-interaction *ε*_ab_. Varying this cross-interaction shows how unfavorable interfaces affect the solvent fraction (*ϕ*_s_) in the brush.

3. Absorption isotherms. To investigate the sorption behavior at different vapor pressures, we use the interaction parameters of the first set of simulations and vary the vapor pressure in order to generate absorption isotherms for all two-component brushes as well as a homopolymer brush with similar chain length and grafting density.

## 3 Results and discussion

In this section, we present the results of the simulations described above. First, we discuss the effect of the copolymer architecture on the structure of the polymer brushes, and the uptake and distribution of solvent throughout the different systems. Next, we show how the solvent uptake depends on the interfacial energy between polymer phases by presenting simulation results at different values of the interaction strength between the two monomer species. Finally, we present absorption isotherms for brushes of various copolymer architectures, as well as mixed homopolymer brushes. These results are compared to the pure homopolymer brush to identify the most promising structures and conditions for enhanced vapor sorption using copolymer brushes.

### 3.1 Interfacial effect

**Fig. 2:**
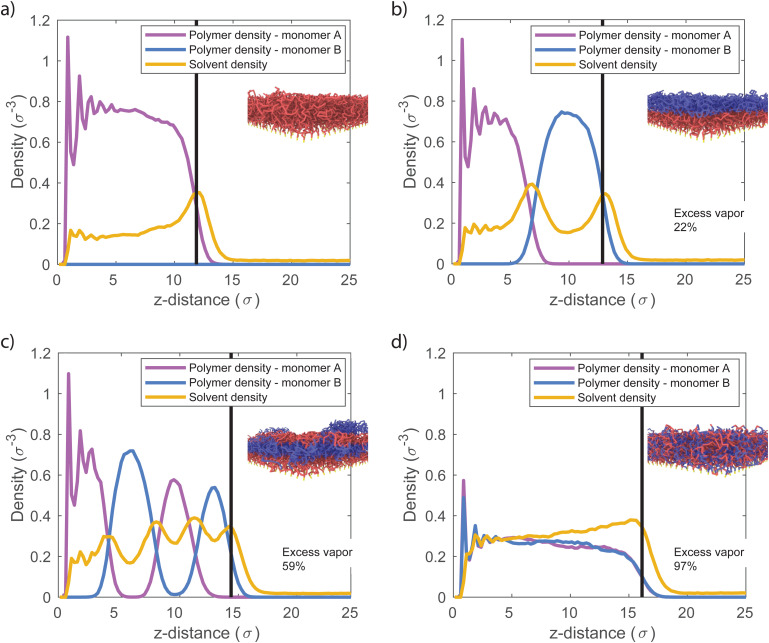
Monomer A (purple), monomer B (blue) and solvent (yellow) density profiles where the black vertical line represents the brush height. Excess vapor accumulates at unfavorable interfaces between immiscible blocks. (a) Pure homopolymer brush, (b) 2-block brush, (c) 4-block brush, (d) random brush. In all simulations we use *ε*_ab_ = 0.4, *ε*_aa_ = *ε*_bb_ = *ε*_as_ = *ε*_bs_ = *ε*_ss_ = 1.

Enhanced vapor sorption in mixed polymer brushes is caused by vapor uptake at high-energy interfaces between the polymeric phases.^29^ We therefore expect that the distribution of the different species will be critical to understand vapor sorption in our block copolymer systems as well. In Fig. 2, we plot the concentration of monomer type A (purple), monomer type B (blue) and the solvent (yellow) as a function of the distance from the grafting plane for the pure, 2-block, 4-block and random copolymer brushes. In these systems, *ε*_ab_ = 0.4, and the pressure of the solvent vapor corresponds to 62% of the saturation pressure. Since *ε*_ab_ < *ε*_pp_, contacts between the two polymer species are unfavorable. In the block copolymer brushes (Fig. 2b and c), these unfavorable interactions result in vertical phase separation, which is in line with earlier simulations of diblock copolymer brushes.^52,53^ This vertical structure can be seen in the density profiles as well-defined peaks in the concentration of monomer species A (purple) and B (blue). The surface-anchored section of the block copolymer chains consists of monomer A, which forms the layer closest to the substrate as a result. In this system, where the monomer species are immiscible, the interfaces between the different polymer layers are narrow and sharply defined. However, upon increasing *ε*_ab_, the area of the overlap region becomes larger and larger with the increasing miscibility, as is shown in Fig. S1 of the ESI.† In the random copolymer case (Fig. 2d), the monomer species cannot phase-separate effectively on a large scale, and their distribution over the brush height is the same.

The distribution of solvent throughout the polymer brushes clearly shows enhanced sorption at interfaces. The yellow profile in Fig. 2 shows the solvent concentration as a function of the distance from the grafting plane. In the homopolymer brush, we find a near-constant concentration deeper in the brush, with a peak in the solvent content at the brush–vapor interface. This indicates the formation of an adsorption layer. For a 2-block brush, we observe two maxima in the solvent density. In addition to the adsorption layer at the brush–vapor interface, we find a second maximum inside the brush at the transition from the A to the B block (at *z* = 6 *σ*). This sorption enhancement is a direct result of the incompatibility of the two monomer types, which leads to the formation of a high-energy interface in the dry brush. Solvent adsorption in this region reduces the number of unfavorable A–B contacts, thereby lowering the energy of the interface. An alternative but equivalent interpretation is that the local cohesive energy density in the brush is reduced by the weak A–B contacts, leading to an increased A–B interfacial tension. Since solvent-A and solvent-B interfacial tensions are zero (the solvent is perfectly miscible with either polymer species), drawing solvent to the A–B interfaces then minimizes the system's interfacial energy.

The 4-block brush system behaves similarly to the 2-block system, with maxima in the solvent density at all A–B interfaces. The solvent uptake in random copolymer brushes does not display such well-defined maxima. Since there are no phase separated regions in this system, the solvent density is distributed approximately evenly over the brush height. However, the unfavorable contacts between the polymer segments reduce the average polymer self-affinity compared to the homopolymer case. As a result, the random brush extends further from the surface than the homopolymer brush, and contains significantly more solvent.

Based on these results, we conclude that all systems adsorb vapor at the brush–air interface, and absorb vapor in the bulk of the brush. In the two-component systems (block and random brushes), we also find enhanced adsorption wherever the two monomer species come into contact. Sorption as a result of A–B contacts is denoted as “excess vapor” in Fig. 2. At *P*/*P*_sat_ = 0.619, the 2-block brush contains 22% excess solvent, and the 4-block brush contains 59% excess solvent relative to the homopolymer brush as a result of the additional adsorbing interfaces. Lastly, the random brush contains 97% excess adsorbed vapor. These results further support that the extra sorption is driven by A–B contacts in general, rather than the presence of large-scale A–B interfaces. An additional confirmation for this conclusion is Fig. S2 (ESI†) where we show vapor sorption behaviour of an alternating polymer brush, which shows similar sorption to the random brush.

### 3.2 Monomer affinity effect

**Fig. 3:**
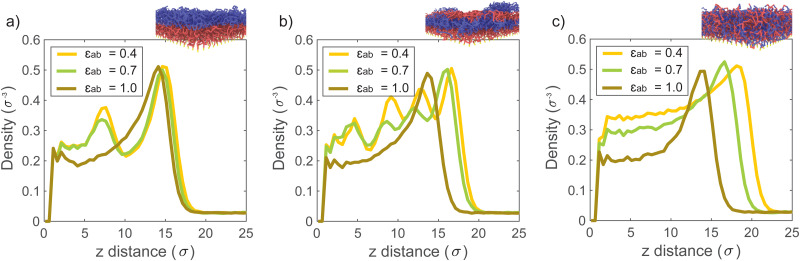
Solvent density profiles at different cross-interaction strengths (*ε*_ab_) for: (a) 2-block brush, (b) 4-block brush, (c) random brush. At low *ε*_ab_ brushes phase separate and excess vapor gets adsorbed at the unfavorable interfaces. Thus, solvent density profiles contain the equal number of peaks to the number of blocks. In a random brush, there is no clear block interface, however, more solvent is sorbed when cross-interactions are unfavorable (at low *ε*_ab_). In all simulations we keep *ε*_aa_ = *ε*_bb_ = *ε*_as_ = *ε*_bs_ = *ε*_ss_ = 1 and only vary *ε*_ab_.

The interfacial energy between the polymeric species depends on the interaction strength between the two monomer types. Hence, we expect this interaction strength to influence the sorption at the polymer–polymer interfaces. In the simulations discussed so far, we set the cross-interaction between the monomer species as *ε*_ab_ = 0.4, and the self-interaction for both monomer species as *ε*_pp_ = 1. Because *ε*_ab_ < *ε*_pp_, the different monomers are immiscible and all two-component brushes self-assemble in such a way to minimize A–B contacts. Here, we present simulation results for which we vary the miscibility of the monomer species, by changing *ε*_ab_. Fig. 3 depicts solvent density profiles for *ε*_ab_ = 0.4, 0.7, and 1.0 in 2-block (Fig. 3a), 4-block (Fig. 3b) and random copolymer brushes (Fig. 3c) with all other parameters unchanged. When *ε*_ab_ = *ε*_pp_ = 1, the monomer species are functionally identical. In this limit, all systems behave like the homopolymer brush, and we find the same solvent distribution in all cases. For *ε*_ab_ = 0.4 and 0.7, where the monomers are poorly miscible, the solvent density profiles once again display maxima inside the block copolymer brushes. The presence of these maxima indicates the presence of the high-energy interfaces between monomer species, which are the result of vertical phase separation in the brush. In the random copolymer brush, where phase separation is not possible, the solvent profile only shows a local maximum at the brush–air interface. With decreasing *ε*_ab_, sorption in the brush increases, as polymer–solvent contacts become more favorable over monomer cross-interactions.

**Fig. 4:**
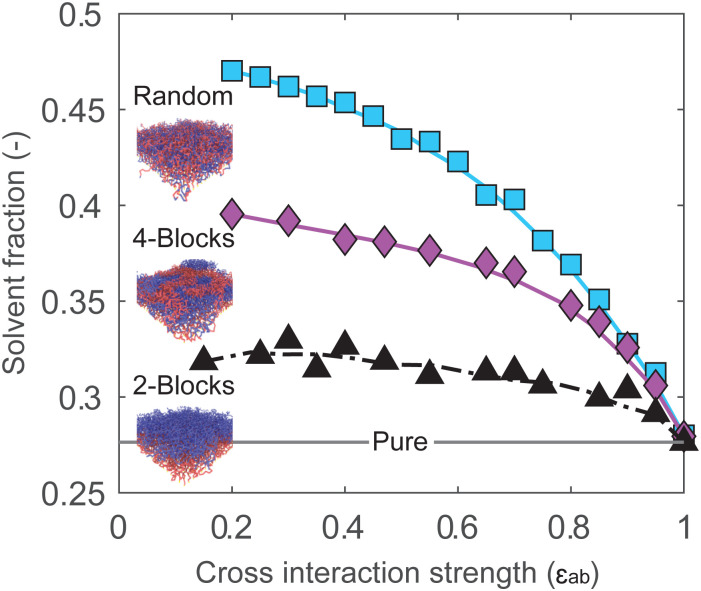
At low monomer cross-interaction strengths (*ε*_ab_), the solvent fraction in the brush is large. When monomers start to mix, at high *ε*_ab_, the enhanced sorption effect disappears for all two-component brushes. For a 2-block brush, dashed line is used to indicate fluctuations in solvent fractions. In all simulations we keep *ε*_aa_ = *ε*_bb_ = *ε*_as_ = *ε*_bs_ = *ε*_ss_ = 1 and only vary *ε*_ab_.

The cross-interaction strength affects the amount of absorbed solvent in the brush. Fig. 4 displays the solvent volume fraction in the brush as a function of *ε*_ab_ for block and random copolymer systems. As previously, at *ε*_ab_ = 1 all systems are effectively homopolymer brushes, and absorb the same amount of solvent. In all systems, reducing the miscibility of the monomers through *ε*_ab_ leads to an increase in solvent uptake. The solvent uptake appears to increase monotonously with decreasing *ε*_ab_, trending towards some saturation value at low *ε*_ab_. The random copolymer brush absorbs the most solvent, followed by the 4-block brush and the 2-block brush, with the largest differences for highly immiscible systems. This is consistent with the expected behavior: sorption is driven by the unfavorable interaction between monomers, and increases with the number of contacts between the two monomer species.

### 3.3 Isotherms

**Fig. 5:**
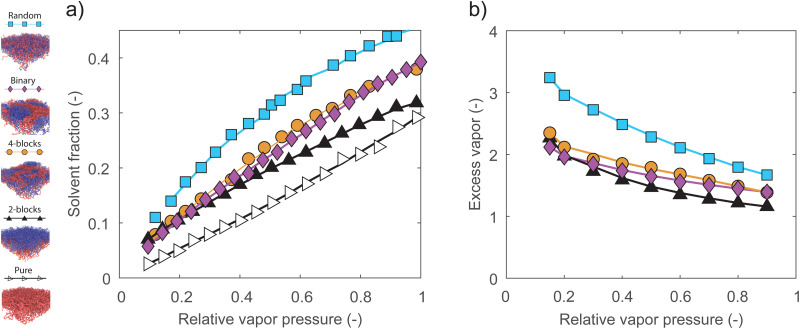
(a) Absorption isotherms of random, mixed-binary, 4-block, 2-block and pure homopolymer brushes. All isotherms appear concave-downward, apart from the convex-upward shape of a pure homopolymer brush. (b) Excess vapor sorbed at different relative vapor pressure in structurally distinct polymer brushes compared to the pure homopolymer brush. The random brush shows significant sorption enhancement at low relative vapor pressures. In all simulations we use *ε*_ab_ = 0.4, *ε*_aa_ = *ε*_bb_ = *ε*_as_ = *ε*_bs_ = *ε*_ss_ = 1.

In the last set of simulations, we vary the pressure of the solvent vapor and investigate the sorption behavior of pure, mixed binary, 2-block, 4-block and random brushes. The mixed binary brush system consists of equal fractions of homopolymers consisting of each monomer species, and is investigated for comparison to previous work.^28,29^ We consider the solvent fraction as a function of the relative solvent pressure, which we define as the solvent pressure normalized by the saturation pressure of the vapor. In Fig. 5a, we display the resulting isotherms, with the relative solvent pressure on the horizontal axis and the solvent fraction in the brush on the vertical axis. For the pure polymer brush, we find a convex-upward isotherm, which is consistent with typical experimental results.^18,20,54^ However, for all two-component systems, we find a concave-upward isotherm. In previous simulation work,^26^ we have found that this concave-upward isotherm occurs for very strong polymer–solvent interactions. Although uncommon, this type of isotherm is also observed experimentally in extremely hydrophilic systems, such as polyelectrolytes or densely hydrogen-bonding systems.^21,23^ The shift in isotherm shape is driven entirely by the net repulsion between the monomers, as the interaction between the solvent and the two monomer species is the same. We once again find that the sorption depends on the number of A–B monomer contacts, with the random brush absorbing the most solvent out of the two-component systems, the mixed and 4-block brushes absorbing similar, intermediate amounts, and the 2-block brush absorbing the least.

We point out that we likely underestimate the sorption in the 4-block brush at low relative pressures. Under dry conditions, inhomogeneous patches of polymer B make up the topmost layer of the brush, making it impossible to define a single thickness for the whole polymer layer. To obtain a well-defined result, we do not include this topmost block when integrating the density profiles to obtain a solvent fraction.

Fig. 5b displays the same data, normalized by the solvent uptake in the pure brush. This gives us an enhancement factor relative to the homopolymer case. While all two-component brushes display a sorption enhancement by at least 1.5 times over the full range of relative pressures, the enhancement is largest at low relative pressures. Here, the random brush shows a particularly strong enhancement. This suggests that random copolymer brushes may be interesting for sensing applications, where it is often necessary to detect some minority component at a low concentration and pressure. This pressure-dependent enhancement may be explained by the fact that increasing relative pressures drive sorption in the polymer bulk as well as at the interfaces. The number of A–B contacts at the polymer–polymer interfaces therefore decreases with increasing relative pressure, as some fraction of the monomers in the interfacial region is displaced by solvent. This reduction in A–B contacts also reduces the interfacial energy that leads to enhanced sorption, leading to the observed trend.

## 4 Conclusions

Unfavorable interactions between monomers enhance the amount of vapor that a polymer brush can adsorb. These unfavorable interactions can be introduced by grafting the different types of polymers to the same surface to form a mixed brush. However, it is difficult to synthesize a mixed polymer brush system with optimal conditions. Therefore, we investigated polymer brushes made of block and random copolymers of which the different monomers have an unfavorable interaction with each other. We compared four different systems: a single-component (pure) homopolymer brush, a 2-block copolymer brush, a 4-block copolymer brush, and a random copolymer brush.

With molecular dynamics simulations, we make observations that lead to the following three conclusions. First, all copolymer systems show an enhanced adsorption of vapor at the interfaces between AB block in the case of the block copolymer brushes or throughout the brush in the case of the random copolymer brush. Thus, block and random copolymers efficiently introduce strongly adsorbing interfaces between different monomer types in the brush. Second, when varying the cross-interaction strength, we observe that the brushes with more AB-interactions have a higher vapor sorption: AB-interactions drive vapor sorption in two-component brushes. Finally, the absorption isotherms of two-component brushes are concave downward, while the isotherm of the pure brush is concave upward. Hence, two-component brushes show an enhanced sorption, especially at low vapor pressures; the block-copolymer brushes sorb 2× as much vapor as a pure brush and the random copolymer brush 3× as much. This enhancement makes these brushes good candidates for novel sensing and separation technologies where target molecules are present at low concentrations.

## Conflicts of interest

There are no conflicts to declare.

## Supplementary Material

SM-018-D2SM00868H-s001
