# Supplementary material for: Enhanced vapor sorption in block and random copolymer brushes
Source: Soft Matter. 2022 Oct 8;18(44):8398–405. doi: 10.1039/d2sm00868h (PMC9667471; doi:10.1039/d2sm00868h)
Supplement: SM-018-D2SM00868H-s001 [file SM-018-D2SM00868H-s001.pdf]

Electronic Supplementary Material (ESI) for Soft Matter.
This journal is © The Royal Society of Chemistry 2022

Supplementary Information
Enhanced vapor sorption in block and random copolymer brushes

Ivona Glišić, Guido C. Ritsema van Eck, Leon A. Smook, Sissi de Beer*

Sustainable Polymer Chemistry Group,
Department of Molecules Materials,
MESA+ Institute for Nanotechnology, University of Twente,
P.O. Box 217,
7500 AE Enschede,
The Netherlands *

1 Difference in sorption upon changing ϵ_{ab}

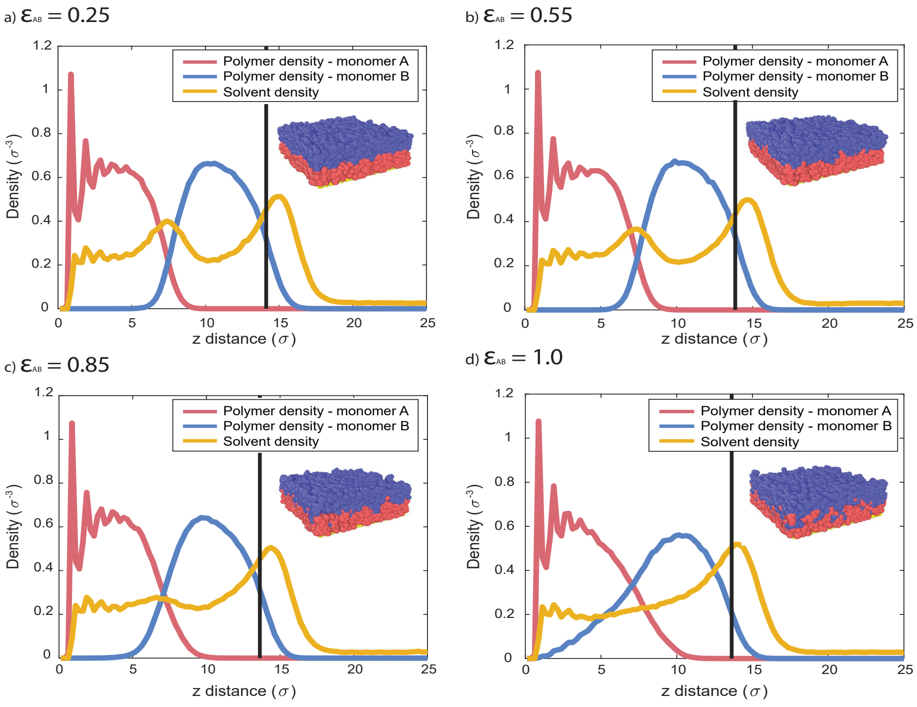

Figure SI 1: Monomer A (purple), monomer B (blue) and solvent (yellow) density profiles at different monomer cross-interaction strengths (ϵ_{ab}). At $\epsilon_{ab} = 1$, the peak correlated to excess solvent disappears due to the mixing of monomers (SI1d). In all simulations we keep $\epsilon_{pp} = \epsilon_{ss} = \epsilon_{ps} = 1$.

*Email: s.j.a.debeer@utwente.nl

2 Alternating brush

We perform additional simulations of an alternating brush (-A-B-A-B-) to investigate the enhanced sorption effect. Even though the alternating brush contains the most A-B interfacial contacts, it does not sorb considerably more vapor than a random brush. To show this, we plot solvent density profiles at $\epsilon_{ab} = 0.4$, 0,7 and 1.0 of an alternating and random brush in Fig. SI 2. Qualitatively, solvent profiles are similar in shape and area. Quantitatively, the difference of solvent sorbed is 0.6 % at $\epsilon_{ab} = 0.4$, 0.4 % at $\epsilon_{ab} = 0.7$, and 0.5 % at at $\epsilon_{ab} = 1.0$. These values are obtained by integrating the areas below the solvent profiles. Therefore, we conclude that there is a negligible difference in vapor sorption between an alternating and random brush.

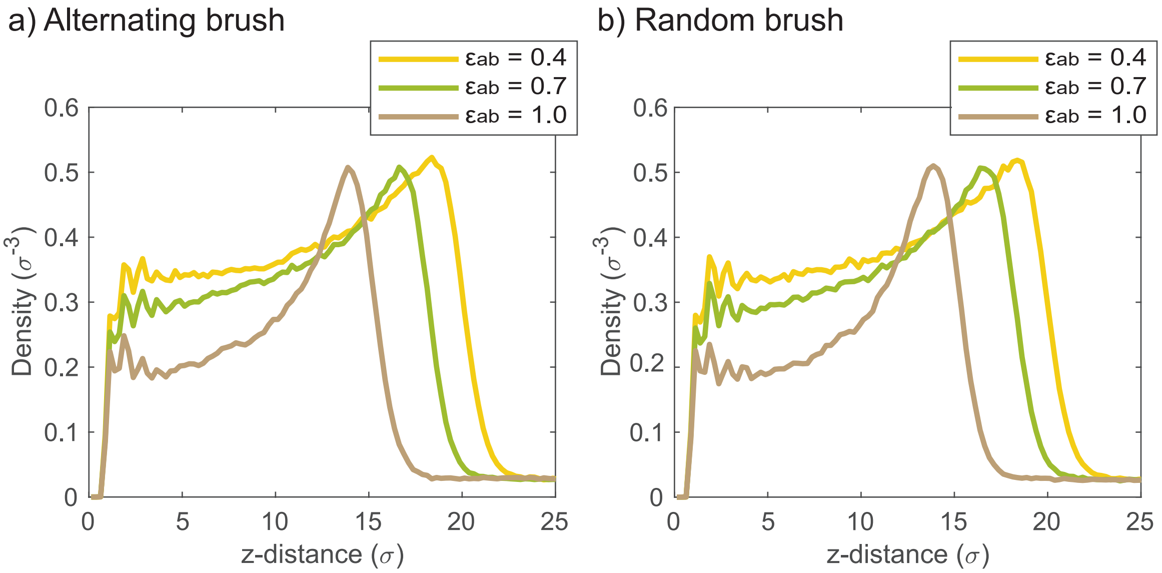

Figure SI 2: Solvent density profiles at different ϵ_{ab} of a) an alternating brush, and b) a random brush. Other particle-particle interactions are kept constant ($\epsilon_{pp} = \epsilon_{ps} = \epsilon_{ss} = 1$).

3 The same particle-particle interactions - the same polymer density profiles

We perform simulations of a pure homopolymer brush and a 2-block brush while maintaining all particle-particle interactions to be 1. When simulating a pure homopolymer brush this means: $\epsilon_{pp} = \epsilon_{ps} = \epsilon_{ss} = 1$. When simulating a 2-block brush, this means: $\epsilon_{ab} = \epsilon_{pp} = \epsilon_{ps} = \epsilon_{ss} = 1$. We observe that the pure brush density profile overlaps with the sum of 2-block brush density profile (sum polymer density A and polymer density B). The polymer density profiles are identical since the particle interactions are identical, as shown in Figure SI 3.

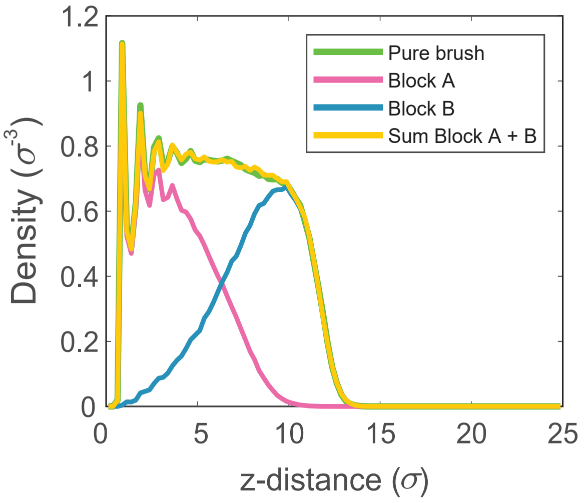

Figure SI 3: Polymer density profile of a pure homopolymer brush (green) and a sum of density profiles in a 2-block brush, A + B, shown in yellow. The profiles are identical since all particle-particle interactions are identical. $\epsilon_{xx} = 1$
